# Editorial: New perspectives on gender based violence: from research to intervention, volume II

**DOI:** 10.3389/fpsyg.2024.1460625

**Published:** 2024-09-16

**Authors:** Luca Rollè, Shulamit Ramon

**Affiliations:** ^1^Department of Psychology, University of Turin, Turin, Italy; ^2^Department of Allied Health Professions, Midwifery and Social Work, University of Hertfordshire, Hatfield, United Kingdom

**Keywords:** DV, IPV, gender base violence, violence, research

The European Institute for Gender Equality and the WHO underlined that Gender based violence (GBV) and Violence Against Women (VAW) involve principally women but also men, families and the societies in which they live REF. The GBV and the VAW reinforce the gender inequalities which are steeped in the cultural aspects and gender roles that either support and justify. In 2015 the EU Agency for Fundamental Rights affirmed that violence against women can be considered as a violation of human rights and dignity REF. In the European Union one in three women experience physical and/or sexual violence, while one in two experience sexual harassment, one in 20 has been raped, and one in five has experienced stalking. Ninety-five percent of victims trafficked for sexual exploitation in EU are women. GBV and VAW can manifest indifferent ways, from the most common forms (i.e. Intimate Partner Violence), to the newer forms mediated, for example, by the use of new technology (i.e. the use of devices and online mode). However, Donestic Violence (DV) remains largely under-reported due to fear of reprisal by the perpetrator, hope that DV will stop, shame, loss of social prestige due to negative media coverage: 90% of cases of DV continue to be identified as a non-denounced violence. Violence toward men should also not be neglected, as it increases in number every year. Its dynamics may differ, and most of it is generated by other men in same sex couple.

## The themes

The Research Topic comprises 15 articles, both theoretical reviews and original empirical research articles. The prevalent themes of the articles in this 2nd volume are much more wide ranging than they were in the first issue. They include the impact of the COVID-19 pandemic on same sex couples (Pistella et al.) and romantic attachment of same sex couples (Tognasso et al.), patterns of ethnic minorities representation in the context of domestic abuse (Bland et al.), risk abuse of intimate partner leading to femicide (Garcia-Vergara et al.), the decision making process of separating from an abusive partner (Di Basilio et al.), opportunities reduction of abuse (Schaefer et al.), hair regulations in prison and its relationship as an abusive strategy (Lo et al.), and the trivialization of verbal abuse of women in India (Arya and George).

More articles focus on GbV toward women than toward men, representing the findings that abuse of women continues to be much higher than the abuse of men, either via domestic or non-domestic abuse.

A range of countries have been looked at, such as Hong Kong, India, Italy and UK, offering an international perspective.

## Methodology and methods

A large number of articles apply mixed methods of both qualitative and quantitative analysis. The qualitative research methods include interviewing victims and at times also perpetrators, and in one case using semi-autobiographical books as source of qualitative data. Qualitative data was analyzed by the application of a variety of thematic analysis. Quantitative methods were based on analyzing data retrieved via a variety of questionnaires, then submitting the data for statistical analysis, inclusive of its reliability and validity dimensions.

## Key findings

Several articles look at the problem areas which lead to abusive behavior by perpetrators, and lack of resistance by the victims, such as poor self esteem by gay people, perceiving women as one's property, a higher rate of IPV among minority ethnic groups who are discriminated against by the majority in a variety of ways, the internalization of guilt by the victim, social trivialization of verbal abuse which is internalized.

Some articles point out to the complexity of key decisions, such as leaving the abuser by the victim, where issues of securing physical and financial living conditions, the welfare of children, the reluctance to give up the notion of romantic attachment, and the social shame play a part.

The role of supporters—be they professionals or family members or friends—seems to be central in providing assistance to victims in this long process.

Focus on reducing opportunities for domestic abuse among probationers and parolees is looked at in one article, as preventive measure.

While most of the studies focus on female victims, one study looked at the use of hair as a tool of aggression in male and female prison in Hong Kong, and concluded that this is the case in male prisons but not in female prisons. Satyen et al. highlights the experience of DV in the Indian context using an intersectional feminist framework analyzing the variables of experience abuse and controlling behavior in relation to the patriarchal belief of the abused women. The result of the study, done with 825 Indian women, underlines the need to involve males in Indian communities through culturally customized intervention tactics aimed to contrast the patriarchal attitudes that spread, rationalize, and justify domestic violence.

The lack of studies on the function of the victims' emotional characteristics, as well as the failure to identify re-victimization by the same or other aggressors, are two of the key shortcomings discussed by Bellot et al.. The results suggest that being the victim of several incidents of IPV perpetrated by various perpetrators can be considered a severe type of re-victimization that impacts poor emotional control and a lack of social support.

De Gregorio et al. investigate the representations of crimes and the social actors engaged in cases of feminicide. The authors discussed the media representations as it has been transmitted by some television programs, emphasizing on narrative profiles of victims, perpetrators of crimes, and their connections. The descriptions are based on the news aired on television, as well as a variety of responsibilities that are related to them by kinship, acquaintance, or professional affiliation.

The work of Davies et al. offer an analysis of all rape and serious sexual offenses (RASSO) committed over a 3 years period in one English Police Force. The result of the study highlights that the response of the police was inadequate seen that many victims reported feeling to be unable to go to the police to report the crime: the article underline the necessity to increase the criminal justice system to give the opportunity to the victims to report the any RASSO.

Zara et al. started from the limited comparative information on the various forms of violence involved, as well as a lack of study on sexual femicides, to investigate incidents of violence against women in northern Italy and in particular they dis concentrating on sexual and non-sexual femicides and comparing them to rape that does not result in femicides. The results suggest that sexual femicides primarily involved unknown victims or prostitutes. Sexual femicidal criminals utilized inappropriate weapons to murder their victims, acted in hidden settings, and left the crime scene; their crime was more often the outcome of predatory intents, with antisociality and sexual deviance being the most prominent elements associated with this form of femicide.

The goal of the article of Cabras et al. was to evaluate the link between sex, gender roles, and the approaches to control assertive and non-assertive responses in a sample of university students. One thousand four hundred fifteen students filled out the questionnaire and the results highlight the need of the Academic Institution to create a easier procedure to facilitate the report and support the victims too. A gender quality plan in Academia can help to develop a non sexist environment for people.

The work of Howard-Merril et al. discussed the LINEA intervention development process with the support of the 6SQuID framework. The results highlight the importance to develop intervention method and to adopt an iterative and fexible approach.

The article of Tognasso et al. analyse the influence of romantic attachment on Same Sex Intimate Partner Violence in a Lesbian Women Italian Sample. Three hundred twenty lesbian women filled in the questionnaire. The findings demonstrated a positive correlation between attachment anxiety and general and psychological same sex intimate partner violence perpetration. Similarly, there was a positive correlation found between attachment avoidance and general, psychological, and physical SSIPV perpetration.

